# The Dark Side of Top Level Sport: An Autobiographic Study of Depressive Experiences in Elite Sport Performers

**DOI:** 10.3389/fpsyg.2016.00868

**Published:** 2016-06-07

**Authors:** Hannah J. H. Newman, Karen L. Howells, David Fletcher

**Affiliations:** ^1^School of Sport, Exercise and Health Sciences, Loughborough UniversityLoughborough, UK; ^2^Faculty of Education and Language Studies, The Open UniversityMilton Keynes, UK

**Keywords:** athletes, depression, health, mental, performance, well-being

## Abstract

The general and sport psychology research converge to point to a complex relationship between depressive experiences and human performance. The purpose of this study was to explore the depressive experiences of top level athletes and the relationship of such experiences with sport performance. Twelve autobiographies of elite athletes representing eight sports were analyzed. The autobiographical analysis was informed by narrative tradition, using three types of narrative analysis: categorical content, categorical form, and holistic content. The analysis revealed a temporal aspect to the depressive experiences that the athletes reported. Initially, sport represented a form of escape from the depressive symptoms which had been exacerbated by both external stressors (e.g., experiencing bereavement) and internal stressors (e.g., low self-esteem). However, in time, the athletes typically reached a stage when the demands of their sport shifted from being facilitative to being debilitative in nature with an intensification of their depressive symptoms. This was accompanied by deliberations about continuing their engagement in sport and an acceptance that they could no longer escape from their symptoms, with or without sport. The findings extend the extant literature by suggesting a reciprocal relationship between depressive experiences and sport performance, and they support the general psychology literature relating to the negative impact of depression on performance. The applied implications of these findings are discussed emphasizing the importance of early identification of depressive symptoms and the adoption of a proactive approach in the prevention and management of symptoms.

## Introduction

World Health Organization [WHO] (2015) reported that an estimated 350 million people were affected by depression. Depression is the term most commonly used to refer to a significant (or clinical) depressive disorder, defined by the Diagnostic and Statistical Manual of Mental Disorders (DSM-5) as the occurrence of one or more major depressive episodes. These episodes are characterized by the presence of five or more depressive symptoms for a period of at least 2 weeks (American Psychiatric Association [APA], 2013). Symptoms identified by the DSM-5 include individuals experiencing a depressed mood, diminished interest or pleasure in activities (anhedonia), feelings of worthlessness or excessive or inappropriate guilt, fatigue or lack of energy, difficulty concentrating, and recurrent thoughts of death or suicide (American Psychiatric Association [APA], 2013). An important consideration within the general rubric of depression is the distinction between a clinical disorder and subclinical states (Parker et al., 2015). Although some symptoms such as depressed mood and lowered self-esteem are core symptoms common to both clinical and subclinical depression, other symptoms such as anhedonia have shown greater specificity to clinical depression (Parker and Paterson, 2015).

The rising economic cost related to depression in the working population is so significant that it now constitutes a significant public health problem (Harvey et al., 2009, 2011). In conjunction with the findings that major depression is more consistently related to poor work performance than any of the commonly occurring chronic physical conditions identified in a working population (Wang et al., 2014), researchers in occupational psychology are increasingly interested in the extent to which there may be a relationship between depression and performance. Using an online measure, Harvey et al. (2011) found that depression may have an impact on occupational performance, in that it is related to reduced concentration, fatigue, disturbed sleep, and poor motivation. Findings from other research have identified that depression is significantly related to decrements in dimensions of work performance such as task focus and productivity (Wang et al., 2014). When compared to depression-free healthy control participants, individuals with depression had significantly greater deficits in managing mental-interpersonal, time, and output tasks, and importantly, this impact persisted even after symptoms had improved (Adler et al., 2006). Both the duration of depressive episodes and the severity of depression are salient in addressing the impact that the mental health issue has on performance in a working environment. The duration of depressive episodes has been linked to the level of functioning and work ability, with longer durations being associated with lower levels of functioning and a higher level of dropout from work (Riihimäki et al., 2015). Although decreased productivity is evident in all individuals experiencing depression, there is a positive correlation between severity and work productivity (Jain et al., 2013).

In the sporting domain, physical injuries and illnesses have typically attracted more attention from athletes, coaches, physicians, and support staff than depression and other mental disorders (cf. Frank et al., 2013). This imbalanced focus is thought to be largely due to the existing social stigma surrounding psychiatric illness (Glick and Horsfall, 2009). However, athletes are arguably exposed to similar stressors and adversities as the general population with sport performers experiencing bereavement, relationship breakdowns, and illnesses (cf. Howells and Fletcher, 2015) which may be related to depressed mood and depressive symptoms. In a review of the depression in sport literature, Wolanin et al. (2015) remarked that “clearly depression in athletes exists. Suicide in athletes, a tragic outcome that can be associated with depression, exists” (p. 59). Accordingly, given the prevalence of depression in the wider community, it is of interest to performance directors, national governing bodies, and coaches to ascertain whether the depression incidence in the athletic population is comparable to the general population or whether there are any significant differences. However, due to inconsistent methodology, erratic reporting, and a poor understanding of tools to evaluate athletes, the underreporting of mental health in athletes remains “a significant concern” (Rao and Hong, 2016).

The majority of the extant research that has explored depression in sport has focused on its prevalence in youth, collegiate, and university populations, particularly from within the United States collegiate sport system (e.g., Proctor and Boan-Lenzo, 2010). This work has tended to compare student athletes and student non-athletes to ascertain differences in prevalence between these two populations. Yet, as a body of literature, the findings are inconsistent with some studies reporting a lower prevalence of depression in student athletes than non-athletes (e.g., Armstrong and Oomen-Early, 2009; Proctor and Boan-Lenzo, 2010) and others reporting a comparable prevalence rate (e.g., Storch et al., 2005; Yang et al., 2007). Interestingly, both Storch et al. (2005) and Yang et al. (2007) found differences in gender, with female athletes reporting more depression symptoms than male athletes and male and female non-athletes. Other student athlete research has examined the difference in depression prevalence between current student athletes and retired student athletes (Weigand et al., 2013) and found that depression was significantly higher in current student athletes than those who had graduated.

In a focus on the elite environment, Mummery (2005) directed attention to depression in Olympic athletes in an essay that referenced Olympic champion, Kelly Holmes,’ experiences of depression. He posited that:

Athletes may be more predisposed than the general population to depression, because of the physical and psychological demands placed on them by the sporting environment. Stress… is associated with depression and is inherent in the life of an athlete (p. S36).

In a review of the extant literature, Frank et al. (2013) highlighted a tension present in elite sport about how depression is perceived. The authors noted that high level athletes are highly vulnerable to developing depressive symptoms due to their status and the extreme pressure that they experience, yet conversely, they are considered especially resilient and therefore are less vulnerable to mental health issues. Several empirical studies have focused specifically on elite sport, Hammond et al. (2013) examined the relationship between the prevalence of diagnosed failure-based depression and self-reported symptoms of depression within a sample of 50 elite swimmers. The study identified a 68% lifetime prevalence of depression episodes among the participants, with significantly more females endorsing history of depression. In an examination of German elite athletes, Nixdorf et al. (2013) found that 15% reported depressive symptoms and also revealed higher levels of depressive symptoms among the individual athletes than the team athletes. More recently, in a cohort of Australian elite athletes, Gulliver et al. (2015) found that approximately a quarter (males = 23.6%, female = 30.5%) of the elite athletes in their study scored above the caseness cutoff score for depression suggesting the presence of a possible depressive disorder. Collectively, this research indicates that depression is prevalent in elite sport as well as student sport.

Although the findings from non-elite sport are equivocal with some research identifying that prevalence is comparable to the wider population (Storch et al., 2005; Yang et al., 2007) and other research finding that depression is less prevalent (Armstrong and Oomen-Early, 2009; Proctor and Boan-Lenzo, 2010), the findings from elite sport research are unequivocal. These differences may be mediated by two main factors: namely, the existence of a dominant narrative in elite sport which involves a stigma associated with depression, and the presence of both risk and protective factors for athletes. The findings from a number of studies (e.g., Glick and Horsfall, 2009; Proctor and Boan-Lenzo, 2010) have suggested that depressive symptoms may be underreported in athletic populations due to the stigma of mental health issues prevalent in the athletic environment. This stigma, alongside negative past experiences of help-seeking and a lack of mental health literacy, constitutes a significant barrier to mental health help-seeking in young elite athletes (Gulliver et al., 2012). Furthermore, there may be a perception that elite athletes are immune to mental illness (Hughes and Leavey, 2012), a perception which has forestalled a need to research both the incidence and etiology of mental illness in elite sport.

There are a number of risk factors for depression in athletes which include being elite (e.g., Hughes and Leavey, 2012; Hammond et al., 2013), injury (e.g., Appaneal et al., 2009; Putukian, 2015), overtraining (e.g., Purvis et al., 2010), identity foreclosure (e.g., Hughes and Leavey, 2012), the pressure to deliver peak performance (e.g., Weigand et al., 2013), sport specific demands (Nixdorf et al., 2015), multiple concussions (e.g., Guskiewicz et al., 2007; Didehbani et al., 2013) and retirement from elite sport (e.g., Lavallee and Robinson, 2007; Hughes and Leavey, 2012; Wolanin et al., 2015). Conversely, a few studies have reported that engagement in sport could also be a protective factor against depression for both males and females (Gore et al., 2001). Research investigating adolescents participating in sport found that as sport participation increases, the probability of suffering from depression reduces by 25% (Babiss and Gangwisch, 2009). This may be explained by the notion that engagement in sport may provide a release of stress (Proctor and Boan-Lenzo, 2010), or it may serve to boost self-esteem and feelings of social connectedness (i.e., social network and team support; Armstrong and Oomen-Early, 2009).

With the majority of sport research, including that which has investigated elite cohorts, focusing on prevalence rates and potential risk and protective factors, there has been limited research investigating the association between depression and sport performance. This is in spite of Mummery concluding in his 2005 essay in *The Lancet* that “the facts remain that any level of depression will affect performance and that the issue of depression in this population should, therefore, be taken seriously by the research community” (p. S37). In a review of the literature, Wolanin et al. (2015) identified that athletes may be susceptible to depressive symptoms when their athletic performance declines or they experience catastrophic choking. This link with performance was identified in a study that investigated mood states following a win, a loss, or a draw in hockey and soccer players and identified that depressive symptoms are related to the failure to achieve performance goals (Jones and Sheffield, 2008). This finding was also reported when an elite cohort was investigated; Hammond et al. (2013) identified a significant relationship between the athletes’ depression symptoms and performance. This study illustrated that elite athletes may be more susceptible to depression when faced with poor performance outcomes. However, the focus on elite sport is still relatively limited and Nixdorf et al. (2013) has posited that more research into the origin and context of depression in elite athletes is needed before helpful interventions can be developed.

Given that elite athletes comprise a population in which possible risk factors for depression, such as performance failure, can be deemed as being salient, the purpose of this study was twofold: firstly, to investigate elite sport performers’ depressive experiences and, secondly, to explore the relationship between these experiences and their sport performance. It is hoped that this study will contribute detailed information and context that has been identified as lacking, with a particular focus on the implications of depressive symptoms for performance, and the implications of performance for depressive symptoms.

## Materials and Methods

### Autobiographical Research

Our individual and social histories are articulated in the act of storytelling, and human beings are storytellers by nature. Through autobiographies individuals provide a written narration of salient aspects of their own lives within the context of the society in which they live, so providing insight into both the individual and the social experience. The use of autobiographies, biographies, and published memoirs as analytical resources to access the stories that people tell has emerged due to a need to expand methodological options in the field beyond (post) positivistic or (neo) realist forms of inquiry (Smith and Sparkes, 2009). As an alternative to other qualitative data collection methods, the participant-led nature of autobiographical accounts means that the narrative proffered is salient to the author in the context of their individual and cultural experiences. The stories that people tell are subject to powerful cultural messages that are representative of a specific time and space. The authors and their autobiographies are not immune to these messages and are not only influenced by, but also serve to reinforce and promulgate, these cultural narratives. Accordingly, published autobiographies constitute social and cultural products that are reflective of the historical era in which they are written (Crossley, 2000).

Taylor (2008) remarked that autobiographies written by sport performers, managers, and officials are numerous, outnumber journalistic and academic studies, and are amongst the most popular of celebrity memoirs. Despite sport scholars using autobiographies in their research for some time now (cf. Howells and Fletcher, 2015), it wasn’t until the publication of Howells and Fletcher’s (2015) and Morgan et al. (2015) studies on psychosocial aspects of elite sport that autobiographies became evident in the extant sport psychology literature. In their paper, Howells and Fletcher (2015, p. 46) provided a rationale for the appropriateness for using autobiographies to address their research question and concluded that the analysis of such sources “provide valuable and privileged insights into psychosocial processes and changes.” Building on the case made in Fletcher and colleagues’ original studies, Sparkes and Stewart (2016, p. 123) remarked that “the analysis provided by Howells and Fletcher makes a positive contribution to our knowledge in SHE [sport, exercise, and health]” and reinforced their argument for taking sporting autobiographies seriously as an analytical resource in sport psychology research. Due to the sensitive nature of depression, the reluctance of elite populations to disclose sensitive information to a stranger (Parsons et al., 1993), and the involvement of the second and third authors who have experience in analyzing sporting autobiographies (cf. Howells and Fletcher, 2015; Morgan et al., 2015), autobiographies were selected as the most appropriate data for analysis. Paradoxically, despite the stigma of poor mental health that exists in elite sport and results in the underreporting of depression, accounts from elite athletes detailing their experiences of depression *are* commercially marketable. So against a backdrop of stigma, the potentially cathartic and therapeutic capacity of writing about depression by athletes competing at the highest level is not only accepted, but publishable and in demand.

### Sampling Procedure

To collate the sample, initially the first author carried out a hand search of elite athletes’ published autobiographies to identify those that had the potential for inclusion. Then, a single-question survey was sent to 30 sport psychology professionals across the United Kingdom requesting that they identify elite athletes’ autobiographies published between 2003 and 2013 which they perceived had documented experiences of depression. Responses to the survey (*n* = 17) were collated and added to the autobiographies identified by the researchers, providing a total of 19 books. Purposive sampling, used in qualitative research to provide information-rich, relevant sources that usefully represent the topic for exploration (Morse, 1991), was then used to determine which autobiographies would be included in the final sample. This allowed a sample to be selected that encouraged a deep understanding of the depression experiences of elite athletes (cf. Maykut and Morehouse, 1994).

A criterion-based form of purposive sampling was used. This is a sub-type of purposive sampling in which the researcher predetermines a set of criteria for selection (Patton, 1990). The initial criterion for inclusion in the sample was that autobiographies should have been published since the turn of the century, a post 9/11 era and one in which “stories of suffering and survival sell to readers” (Schaffer and Smith, 2004, p. 12). The second criterion was that the autobiographies were those of elite athletes. Athletes were classified as ‘elite’ using the continuum of validity proposed by Swann et al. (2015). In this case, athletes were considered elite if their highest standard of performance was international level, national level, top tier, or second tier professional leagues. The third criterion was that the autobiographies documented detailed experiences of depression. Experiences of depression were identified using the criteria and symptoms identified by the DSM-5 (American Psychiatric Association [APA], 2013) and the recent literature on depression (Parker and Paterson, 2015; Parker et al., 2015), including persistent depressed mood, lowered self-esteem, and anhedonia. For the purpose of this study, clinical diagnoses of depression were included as well as any experiences that the athletes identified as either depression, or a depressive episode.

Exclusion criteria were also applied to the original sample. Autobiographies that comprised athletes’ reports of detailed periods of emotional distress, but did not identify them as depression, were excluded (e.g., McGuigan, 2011). Those narratives that primarily focused on addiction as a consequence of depression, with little detail provided on the symptoms and experience of depression, were also excluded from the study (e.g., McGrath and Hogan, 2006), as were those which did not provide in-depth detail of the experiences (e.g., Wiggins and Gallagher, 2008). Finally, autobiographies were also excluded if the athlete disclosed clinical diagnosis of another psychological disorder which could impact upon their experiences (e.g., Collymore and Holt, 2004).

Following application of the inclusion and exclusion criteria, twelve autobiographies were selected, a number that is comparable with previous multiple sport-related autobiography studies (viz., Stewart et al., 2011; Howells and Fletcher, 2015; Morgan et al., 2015). The sample (see **Table 1**) comprised the autobiographies of nine male, and three female athletes who represented three nationalities, and competed in eight sports (boxing, cricket, cycling, football/soccer, rugby union, snooker, swimming, and tennis).

**Table 1:** Athlete and autobiography details.

Athlete	Sport	Nationality	Title	Publication year	Co-author
Graeme Obree	Cycling	British	The flying Scotsman	2003	Not applicable
Marcus Trescothick	Cricket	British	Coming back to me	2008	Peter Hayter
Andre Agassi	Tennis	American	Open	2009	J. R. Moehringer
Serena Williams	Tennis	American	My life: Queen of the court	2009	Daniel Paisner
Graeme Dott	Snooker	British	Frame of mind	2011	Derek Clements
Jonny Wilkinson	Rugby Union	British	My autobiography	2011	Owen Slot
Leon McKenzie	Soccer	British	My fight with life	2012	Alan Swann
Victoria Pendleton	Cycling	British	Between the lines	2012	Donald McRae
Ian Thorpe	Swimming	Australian	This is me	2012	Robert Wainwright
Amanda Beard	Swimming	American	In the water they can’t see you cry	2013	Rebecca Paley
Clarke Carlisle	Soccer	British	You don’t know me but… A footballer’s life	2013	Ian Marshall
Ricky Hatton	Boxing	British	War and peace: My story	2013	Tris Dixon

### Data Analysis

Given the relative infancy of using autobiographies in sport psychology research, there is an absence of methodological guidance on how to analyze the data. Informed by the narrative tradition, we adopted a combination of both thematic and structural analyses (cf. Sparkes and Stewart, 2016) that was similar to the process employed by Howells and Fletcher (2015) in their study of Olympic Champion swimmers. However, unlike the process utilized by Howells and Fletcher which involved two types of narrative analysis: holistic form and holistic content, the present study involved three types of narrative analysis: categorical content, categorical form, and holistic content (Lieblich et al., 1998) with more of a focus on the categorical aspects of the autobiographies. Exploring the autobiographies from a categorical perspective facilitated the dissection of the original stories to identify content relating to depression (cf. Lieblich et al., 1998).

In the first instance, holistic-content analysis was used to analyze the text within the context of the whole life story. Through multiple readings of the autobiographies, patterns were identified to enable exploration of both the internal thought processes and external precipitating factors which may have contributed to the onset of depression or depressive episodes. This analysis enabled an exploration of the potential long-term implications and management of depression. Then, categorical-content analysis focused on the content of the autobiographies in the particular sections of sub-text that detailed experiences of depression, selected via the criteria discussed previously. In accordance with Lieblich et al. (1998), relevant sections of the text were selected and withdrawn from the context of the life story before being subjected to a descriptive content analysis. Sections of text were primarily selected if they contained direct reference to depression or a depressive episode. Other sections of text were also selected if terms other than the word ‘depression’ were used but detailed periods of emotional distress which were either previously or subsequently identified as depression (see **Table 2**). The text selected through categorical content analysis provided the basis for the core themes identified, but to avoid possible fracturing of the data, the quotes presented in the results section were accompanied by the context of the experiences established at the holistic content analysis phase (cf. Iborra, 2007).

**Table 2:** Key terms used for the selection of sub-text during analysis.

Key terms	
Bad place/bad time/bad way	Lonely/loneliness
Black/blackest/blackness	Losing my mind
Bleak/bleakest period	Low/lowest moment/lowest point
Breaking point	My condition
Cry/crying/cried	My illness
Dark/darkest/darkness	Negative/negativity
Depression/depressed/depressing/depressive Desolate	Over the edge Overwhelming sadness
Despondent	Pointlessness
Die/death/dying	Rock bottom
Distress/distressed	Sadness/saddest/sad periods
Down/downer	Suffocating mist
Emotional turmoil	Suicide/suicidal
Empty	Torture/torturous
Gloomy funk	Unable to function
Inner crisis/inner strife	Unmotivated
Isolation/isolated	Warped mindset
Listlessness	Worthlessness

Finally, categorical-form analysis focused on the discrete stylistic or linguistic characteristics of the previously selected sections of sub-text detailing experiences of depression (Lieblich et al., 1998). This analysis was supported by the use of Smith and Watson’s (2010) strategies for reading autobiographies. The autobiographical “I” was a particularly relevant strategy for this study especially in exploring the linguistic strategies used by the authors to detail the depressive experiences of the athletes. All of the autobiographies were written in the first person but as Sparkes and Stewart (2016) postulated, the “I” is neither singular nor first, rather expressions of the self are culturally constructed and represent the articulation of different narratives through which individuals make sense of themselves over time and space. The autobiographical “I” was often self-critical in this study, with a number of the athletes considering themselves outsiders in both their social and professional groups. To elucidate, the self-critical voice in Obree’s narrative was confirmed with statements such as: “I hated myself for what I was” (Obree, 2003, p. 53). Smith and Watson (2010) proposed the exploration of multiple and/or conflicting voices, which can be evident in the autobiographical narrative. In the present study, there was often conflict between the inner voice of the athlete and the ‘socially acceptable’ voice regarding their depression, portraying a sense of ‘what I feel’ versus ‘what I should feel. Agassi stated: “I tell myself that you can’t be unhappy when you have money in the bank… But I can’t help it… I refuse to admit that I feel this way” (Agassi and Moehringer, 2009, p. 232). Furthermore strategies such as the use of metaphor [e.g., “I was lost in a fog of uncertainty” (McKenzie and Swann, 2012, p. 18)], alliteration [e.g., “I was so disenchanted and depressed” (Obree, 2003, p. 107)], and repetition [e.g., “I hate tennis, hate it with a dark and secret passion” (Agassi and Moehringer, 2009, p. 3)] reinforced the traumatic experiences of depression. After each autobiography had been analyzed using these three independent strategies, the sample was considered as a whole by identifying similarities and differences between the patterns and identifying themes emerging from the analysis of the autobiographies.

## Results

The analysis of the autobiographies identified a complex interaction between depression and sport performance, with the varying role of sport affecting both positive and negative consequences for the athletes’ depressive experiences. Episodes of depression had implications for sporting performance, and performance had implications for depressive symptoms. Many of the athletes initially viewed sport as a safe-haven from their depressive symptoms. Enhanced perceptions of this protective feature of their sport was evident following sporting success, but these perceptions were only apparent in the short-term, with symptoms soon returning, and intensifying on occasions of failure. For many of the athletes, when their symptoms were at their most intense, sport no longer provided the escape that it once had. A deleterious impact on performance became apparent, the quote from cricketer Trescothick elucidated the link between emotions, cognitions, and performance as he “tried to find the switch to turn off (his) personal feelings” but couldn’t, and as a result became “very distant and detached” whilst trying to concentrate on his batting (Trescothick and Hayter, 2008, p. 178). The athletes’ inner conflict was manifested in ruminations about the feasibility of their continuation in elite sport. There was an ambivalence evident in the decisions made, whilst the intensified symptoms of depression made it difficult to train and compete, feelings of worthlessness, and craving for further success, seemed to underpin their desire to continue.

### Life Stressors

The athletes often described depressive episodes and emotional difficulties in response to a range of stressors occurring prior to, or during their careers. Their descriptions and use of linguistic strategies such as alliteration and metaphors served to reinforce the traumatic nature of these stressors. Several of the athletes reported traumatic external stressors, such as bereavement. Williams referred to the “agony and anguish of losing Tunde [her sister]” (Williams and Paisner, 2009, p. 165), her use of alliteration in this instance reinforces to the reader the extent of her loss. Dott endured the cancer battle and subsequent death of his long-term mentor, manager, and father-in-law, Alex (Dott and Clements, 2011, p. 155). Beard experienced family troubles following the breakdown of her parents’ marriage, an occurrence that she described as leaving her life “destroyed” following a “perfect” early childhood (Beard and Paley, 2013, p. 22). McKenzie suffered from multiple injuries that meant that soccer was “taken away” (McKenzie and Swann, 2012, p. 21) from him for long periods. Others appeared to have to contend with internal stressors such as their battles with self-loathing and social anxiety. Obree stated that he saw himself as “inherently unlikeable” and adopting alliteration to emphasize the impact that his early experiences had upon his development as an adult, declared that his lonely childhood had left him with “an isolationist and insular personality as well as a real and subconscious fear of social situations” (Obree, 2003, p. 4). Similarly, Pendleton described herself through the use of metaphor as a “seething tangle of doubt and insecurity” (Pendleton and McRae, 2012, p. 3), and termed herself an “outcast” in social situations (Pendleton and McRae, 2012, p. 73). For many of the athletes, these battles with the self were persistent throughout the narrative, conveying a harshly self-critical tone.

### Escapism through Sport

The majority of the athletes viewed their sport as a form of escape from the stressors they experienced, and from the symptoms of depression itself. For some, their pre-career struggles encouraged their early involvement within sport, such as Beard, who stated: “water had become my getaway. The silent sanctuary was my biggest distraction from the troubles with the family” (Beard and Paley, 2013, p. 22). For others, sport continued to be a positive escape following the occurrence of stressors during their career. Dott described each upcoming tournament as “another opportunity to escape from it all” (Dott and Clements, 2011, p. 166), and Thorpe stated that swimming had been his “salvation” (Thorpe and Wainwright, 2012, p. 280). Trescothick found sport to be a relief from the symptoms of depression: “playing top-level cricket gave me such a buzz that I could force them [depressive symptoms] to one side” (Trescothick and Hayter, 2008, p. 23). An exception to this was Agassi, who perceived that he was forced into tennis by his father, and exclaimed that: “I hate tennis, hate it with a dark and secret passion, and always have” (Agassi and Moehringer, 2009, p. 3). His repetition of the word “hate” provided emphasis about the extent to which he viewed the sport.

### Reliance upon Success for Increased Self-Worth

Success in sport provided a positive, but short-lived, impact on the athletes’ feelings of self-worth. Trescothick reported that he: “briefly felt better after making 85 not out” (Trescothick and Hayter, 2008, p. 151). Wilkinson directly declared that success in rugby gave him “a bit of self-worth” (Wilkinson and Slot, 2011, p. 246). Most poignantly, Obree deemed success in an attempt to break the cycling world hour record to be crucial in justifying his very existence even if this voice was outwardly silenced in both his public and private spheres: “If I told them that the hour-record was not about glory… but justifying my next breath, then I would have confused them, to say the least” (Obree, 2003, p. 131).

The athletes articulated that success was important for gaining the approval of others, which in turn was imperative for the athletes’ sense of self-worth. Pendleton described how she could not abandon her bike because she “needed dad’s approval too much” (Pendleton and McRae, 2012, p. 24), a need for approval which continued into her professional cycling career, where she “ached” for the approval of her coach: “hoping that one day I would be good enough to be called Fred’s favorite” (Pendleton and McRae, 2012, pp. 72–73). Hatton emphasized the importance of success for him by branding his comeback fight as being “about redemption and making people proud of (him) again” (Hatton and Dixon, 2013, p. 3) and stated that when suffering from depression “the last thing you want to see is people disappointed in you” (Hatton and Dixon, 2013, p. 271). Sporting success is thus heavily relied on as a way of combatting both the low self-esteem and self-loathing associated with depression.

### Emotional Cost of Failure

In contrast to the positive, albeit short-lived, psychological effect of sporting success, failure had significant and often long-term negative psychological impact upon the athletes. Agassi described one loss as having a “kind of lingering effect” (Agassi and Moehringer, 2009, p. 228) and suggested that the negativity of failure was hard to move on from. Pendleton also described this persistent negative impact post-failure: “It was difficult for them [her teammates] to fathom how desperately upset I became whenever I failed” (Pendleton and McRae, 2012, p. 262). The extent of this negativity was emphasized by Obree, who stated that for him, failure was a case of “emotional death and self-destruction” (Obree, 2003, p. 116). Hatton went as far as to suggest that his sporting failure triggered the beginning of his depressive episode: “When I got beat, that’s when my depression started” (Hatton and Dixon, 2013, p. 190). The intense negativity associated with failure highlights the fragility of the increased self-worth and positive feeling created by success.

The athletes demanded exceptional performance from themselves, and perceived any performance not meeting their exacting and high standards as a failure. Pendleton declared that at the 2008 Beijing Olympics: “anything less than gold, for (her), would define failure” (Pendleton and McRae, 2012, p. 159). For Hatton, a defeat was a failure, regardless of the opponent: “when people said I’d been beaten by the two best fighters in the world it meant nothing to me… I didn’t care who they were” (Hatton and Dixon, 2013, p. 224). This extreme view of success and failure was seemingly underpinned by the athletes’ perfectionist nature. Wilkinson epitomized this character: “my whole life has revolved around… seeking perfection” (Wilkinson and Slot, 2011, p. 248). This perfectionist drive was evident in all aspects of the athletes’ lives, beyond sport, such as Carlisle’s approach to his education: “I can’t just ‘get a degree.’ I am allegedly intelligent, so nothing less than a first will do” (Carlisle and Marshall, 2013, p. 178). Interesting, this quote also suggests the lack of self-belief that underlies the perfectionist tendencies with the use of “allegedly” indicating a failure to internalize this view.

Accompanying this perfectionist drive and the need to succeed, particularly in the eyes of others, was a manifest fear of failure. Dott described fearing looking like “a complete and utter fool” (Dott and Clements, 2011, p. 194), whilst Hatton reported “the fear of being a laughing stock” (Hatton and Dixon, 2013, p. 6). When failure did occur, the athletes reported extreme shame and embarrassment. Carlisle, talking of a play-off final defeat, stated: “the hurt, shame, sorrow, embarrassment, and despair in that moment is quite possibly the worst feeling in football” (Carlisle and Marshall, 2013, p. 268). McKenzie perceived his injury problems as failure, and “was embarrassed because (he) was desperate to show this club how good (he) could be” (McKenzie and Swann, 2012, p. 22). Many of the athletes reported their shame in letting others down through their failure. Beard felt that she had “crushed others’ heartfelt expectations” when her performances declined upon reaching puberty (Beard and Paley, 2013, p. 71), and Carlisle professed that he had “let (his) whole world down” (Carlisle and Marshall, 2013, p. 271).

Sporting failure and the perception that they are perceived as being a failure by others are both evidenced as having substantial implications for athletes who are experiencing, or have experienced depressive symptoms. The need to succeed, fear of failure, and perfectionist drive culminates in extreme negative affect when failure occurs. Interestingly and perhaps in contradiction, some of the athletes acknowledged perfectionism and fear of failure as being crucial to their sporting success. Beard claimed that the perfectionist drive: “made me a star athlete in the water” (Beard and Paley, 2013, p. 89). Wilkinson echoed this: “I still strive to be perfect because it’s what gives me my edge over others on the field” (Wilkinson and Slot, 2011, p. 375). Beard recognized though that although being important to her success in the water, this drive had negative impacts to her wellbeing: “out of the water (it) tore me apart” (Beard and Paley, 2013, p. 89).

### Impact of Depression on Performance

For many of the athletes, their sport had once been a positive form of escape from the emotional difficulties they were experiencing. However, as their depressive experiences intensified, it became apparent that sport was no longer the sanctuary that it had been, and that their performance was negatively affected by their symptoms. For some of the athletes, the pain of their emotions made it difficult to perform optimally. Obree recounted, using powerful imagery that training “seemed like nothing short of bloodless self-mutilation” (Obree, 2003, p. 262). For Trescothick, the symptoms were so intense that he was forced to leave the field of play mid-match during a tour warm-up game in Australia: “I knew it was over. I asked the umpire if I could go off for a leak and I never came back” (Trescothick and Hayter, 2008, p. 316). Other athletes conveyed a defeatist attitude, as though they had given up. Describing one match, Agassi admitted: “I just don’t want it. I know I can beat him and yet it’s not worth the trouble” (Agassi and Moehringer, 2009, p. 160). Some of the athletes observed their anti-depressant medication also having an impact to performance. Trescothick felt that the antidepressants “might be taking the edge off (his) reactions” (Trescothick and Hayter, 2008, p. 286), and Obree made a decision to stop his medication: “I was on 1.2 g of lithium per day, which can have serious side effects – so that and the anti-depressants had to go” (Obree, 2003, p. 280).

### To Quit or Not to Quit?

Several of the athletes conveyed major inner conflict surrounding their involvement with their sport. After long, intense periods of battling with their depressive symptoms, the escapism they achieved through sport was lost. Interestingly, several of the athletes described their struggle to resolve their conflict by engaging in a linguistic strategy that is reminiscent of animism, that is giving inanimate objects life-like qualities; the sports became active ‘participants’ in the athletes’ life choices. Following her sisters death and a return to tennis post-injury Williams professed: “for the first time, *tennis couldn’t solve anything* [emphasis added] for me” (Williams and Paisner, 2009, p. 174), and similarly, for Trescothick, when his symptoms intensified further “the *cricket didn’t seem to help* [emphasis added] like it had in the past” (Trescothick and Hayter, 2008, p. 150). For some, there was resentment toward their sport, suggesting that it was an active contributor to their emotional difficulties. Pendleton’s frustration was apparent: “sometimes I felt it so literally I could have taken a hammer to my bike and smashed it to pieces. I sometimes wanted to quit cycling” (Pendleton and McRae, 2012, p. 80). Obree particularly highlighted this, again using alliteration: “I was so disenchanted and depressed with everything, particularly cycling” (Obree, 2003, p. 107) and considered himself “dead as an athlete” (Obree, 2003, p. 255). However, he “carried on like this for some time, unwilling to let go what still seemed like (his) only hope of self-worth” (Obree, 2003, p. 281).

This need for sport to attain self-worth became more evident for some of the athletes in their retirement, or during periods of not competing. Some of the athletes felt feelings of worthlessness, which contributed to an inner conflict and creating a ‘can’t live with it, can’t live without it’ mentality. Agassi summarized this well when reflecting on his final competitive match at the 2006 U.S. Open: “Please let this be over. I don’t want it to be over” (Agassi and Moehringer, 2009, p. 9). Hatton agonized over the decision to retire following emphatic defeats, and lack of involvement in the sport led to him questioning his identity: “I was rattling about the house, aimless. Everything that I stood for over the last twenty years was gone. I was Ricky Hatton, a boxer” (Hatton and Dixon, 2013, p. 230). The majority of the athletes displayed conflict as to whether continuing in, or retiring from their sport, would help alleviate their depressive experiences. Some realized that success no longer brought them the self-worth that they craved, while others were desperate to recapture that feeling, but were unaware of whether they were still capable of success. This conflict was not evident for McKenzie though, whose time away from soccer was forced upon him through injury, and was perceived purely as damaging to his mental health: “the thought of finishing in football brought me down mentally in an instant” (McKenzie and Swann, 2012, p. 30).

### Challenging Perceptions of Athlete Immunity to Depression

The occurrence of depression was hard for some of the athletes to accept initially, some demonstrated inner conflict between how they felt, and how they thought they should feel. Apparently underpinned by the perception of athletes and successful people being immune to depression and mental illness, some of the athletes, such as Trescothick, articulated their self-critical internal voice: “what the hell did I have to be depressed about? We’d just won the Ashes, life was going pretty well…” (Trescothick and Hayter, 2008, p. 247). Hatton, similarly, stated that “it wasn’t the right way to think” (Hatton and Dixon, 2013, p. 233). Thorpe was concerned about the perceptions of others: “I can imagine how some people, when they look at my life… could say that I have no right to be depressed” (Thorpe and Wainwright, 2012, p. 276). Carlisle described though, how the process of being educated on depression challenged this perception: “Finding out what the illness actually is and how it manifests itself is a process that liberates you from the stigma about the ‘D’ word” (Carlisle and Marshall, 2013, p. 163). A number of the athletes described a similar change in opinion as their mental health literacy increased, leading them to encourage others to change their perception of athlete immunity. Thorpe best depicted this: “it’s another reason why athletes shouldn’t be seen as a robotic group of super humans. We’re all different, each with our own problems” (Thorpe and Wainwright, 2012, p. 279).

## Discussion

Through an analysis of autobiographies we investigated elite sport performers’ depressive experiences and explored the relationship between these experiences and the athletes’ sport performance. This relationship was reciprocal and was characterized by a circuitous association whereby performance impacted on the occurrence of depressive experiences which, in turn, impacted on performance. The integral role of sport, and specifically sport performance, in relation to the depressive experiences was a key feature of the athletes’ narratives and was often characterized by inner conflict about the athletes’ continuation in elite sport typified by a ‘can’t live with it, can’t live without it’ approach. Interestingly, the nature of the role of sport in the athletes’ depressive experiences vacillated between the positive and the negative; on occasions sport had a positive impact on depression and other occasions it had a negative impact. On the latter point, the link with performance was salient with an overreliance on performance success for self-validation leading to extreme emotional costs of performance failure which in turn intensified depressive symptoms. Accordingly, the intensified symptoms following performance failure often had further negative implications for subsequent sport performance. Consistent with the literature that has identified a reluctance to engage with mental health services (Gulliver et al., 2012), help-seeking was delayed by many of the athletes’ as a result of their internalization of the notion that as elite athletes at the height of their careers they should *not* be experiencing depression.

The vacillating nature of the role of sport, along a positive–negative dimension highlighted the complexity of the relationship between sport and depression, and this study partially addresses some of the inconsistencies in student athlete research (Storch et al., 2005; Armstrong and Oomen-Early, 2009; Proctor and Boan-Lenzo, 2010). The cross-sectional design of the previous studies meant that they have identified poor or positive mental health at a specific moment in time, rather than accessing the temporal and dynamic nature of responses about mental health states. It is apparent in the analysis of the autobiographies that self-worth afforded by performance success, and the extreme emotional costs of performance failure, that prevalence rates may be highly affected by recent performance results.

Increased self-esteem was demonstrated in the current study to be a fleeting response to performance success that was ultimately short-lived. This aligns with general psychology literature which regards self-esteem as a relatively stable trait; small changes are possible, but large shifts from relatively low self-esteem to relatively high self-esteem are unlikely (Orth and Robins, 2014). A dominant narrative articulated by the elite athletes was the elevated emotional cost of failure. Although previous research had proposed a relationship between failure and depression (e.g., Hammond et al., 2013), the current study identified that, for elite athletes the need to succeed is integral to their sense of self and was commensurate with their identity as elite athletes, therefore their perceptions of the personal significance of failure was enhanced. The need to succeed was not dependent solely on what was at stake in their professional careers but also what was at stake in their personal lives. The significant negative psychological impact of failure described appeared to form part of a cycle in which the need for success created an intense fear of failure. If failure occurred, depressive symptoms intensified, further driving the need for success to increase self-worth and validate the athletes’ existence. Although failure tended to intensify the symptoms of depression, these symptoms had subsequent negative implications for performance, therefore increasing the possibility of failure. The negative impact of depression on performance found in the athletes emulates that identified in the occupational psychology literature, which has found that the capacity to work is fragmented, with time management, daily work demands, and emotions causing difficulties for depressed individuals (Bertilsson et al., 2015).

In his recent editorial, Bauman (2016) posed the question: “Are mental toughness and mental health seen as contradictory in elite sport (p. 135)?” The answer is, unfortunately, sometimes yes. Confusion occurs when, paradoxically, weakness is misconstrued as strength. There are numerous examples of this elite sport – the promotion of a single-minded ruthlessness, a stubborn bloody-mindedness, conforming to unhealthy behaviors, pushing oneself beyond one’s limits – but one of the most pervasive and damaging instances is that a mentally tough or resilient athlete should deny the stress that they are experiencing and/or not disclosure any mental health issues they are experiencing to avoid appearing weak or vulnerable, despite nothing being further from the truth. Mentally tough and resilient athletes seek social and professional support (cf. Jones et al., 2002; Fletcher and Sarkar, 2012), both proactively in anticipation of challenges and reactively in times of adversity. The promulgation of the ‘macho heroic myth’ of mental toughness and resilience does *not* represent informed, evidence-based understanding or critique of these concepts; rather, it only serves to perpetuate misunderstanding about the complex relationship between performance enhancement and mental health in elite sport. Researchers and practitioners should expose inaccurately labeled ‘mentally tough and resilient behaviors’ for what they really are – weaknesses that will ultimately compromise mental health and wellbeing.

Sport psychologists, coaches, parents, and other key figures involved with elite athletes should be cognizant of the dynamic role that sport may play in relation to experiences of depression. It is apparent that many elite athletes may at various times in their lives use sport as a sanctuary from life’s adversities such as depression (cf. Howells and Fletcher, 2015). Practitioners and significant others should be aware that this tactic does not preclude sport becoming a source of poor mental health particularly in those athletes with a strong athletic identity (cf. Brewer, 1993) who have an intense fear of failure. Depression has been identified as a symptom of overtraining (Purvis et al., 2010), and therefore, in those who appear to immerse themselves in their sport as a form of escape, training load, and volume should be monitored to prevent further negative consequence. They should also take an active interest in the athletes’ lives beyond sport, ensuring that they are aware that support is available for any issues they may have within, or external, to their sport. It is important to note that the symptoms of depression, particularly those that are evident in sport, such as the implications for performance, may not be evident from the beginning of the athletes’ depressive experience. Initially, the escapism that the athletes engaged in had positive consequences which is consistent with Stenseng et al.’s (2012) model of escapism which described using activity engagement as a form of self-development. For some of the athletes in this study, the impact to performance, and the loss of sport as a ‘safe haven,’ only occurred after they had been battling with depression for some time. Therefore it is important for practitioners to be aware of potential pre-cursors, or warning signs, such as an overly self-critical nature, perfectionism, and fear of failure. Measures could be put in place to identify and monitor these signs, as well as the monitoring of responses to stressors, such as performance failure, bereavement, and family problems, as experienced by the athletes in this study.

The publication of Howells and Fletcher’s (2015) and Morgan et al.’s (2015) original analyses of autobiographies in sport psychology research prompted impassioned, yet contrasting, responses (see, e.g., Collins et al., 2016; Day, 2016; Sparkes and Stewart, 2016). For example, Collins et al. (2016) were largely disdainful of the use of autobiographies as an analytical resource and, somewhat inexplicably, quoted purported participants from their study to illustrate and emphasize their points. In contrast, Sparkes and Stewart (2016) presented a zealous promotion of autobiographies in sport-related research, arguing that “negative views and misplaced assumptions…. are somewhat exaggerated… [and that] our presentation in this article has been necessarily celebratory of possibilities rather than critical of actualities” (pp. 115, 119, 127). We prefer to adopt the balanced stance taken by Howells and Fletcher (2015) that autobiographies *do* provide a source of valuable information for researchers seeking to better understanding psychosocial aspects of sport, *but* that for a variety of reasons such sources need to be critically analyzed and interpreted (like any other type of data or documents; see also Morgan et al., 2015). Moreover, perhaps a more fundamental point worth emphasizing is that qualitative research is by its very nature subjective (cf. Denzin and Lincoln, 2011), so attempts to enforce epistemological or procedural impositions on researchers is, in our opinion, contrary and counterproductive. Rather, the choice of methods, analysis and interpretative approach adopted should embrace (qualitative *and* quantitative) diversity and in large part be driven and judged by their appropriateness to answer the research question(s) posed (cf. Hardy et al., 1996; see also Fletcher and Wagstaff, 2009; Hardy, 2015); as Fletcher and Arnold (2016) recently remarked, “in any area of psychosocial research, methodological partiality based on erroneous dogmatism should not… dictate the selection of methods in preference to their appropriateness for addressing original and significant research questions” (p. 17). The decision to use (or not to use) and how to use autobiographies in research should be no different in this regard.

Future research investigating depression in elite athletes should further examine the complex relationship between depression and performance. Of particular interest are the specific antecedents or mechanisms that accompany the transition from sport as a safe haven to sport being a source of a deleterious emotional response for those who have experienced depression or are susceptible to depressive experiences. Further research is also needed regarding the inner conflict that athletes experience when they are unable to alleviate their symptoms through continuing their sport or through retiring or taking a break. This is important because it is in this stage, when sport no longer constitutes an escape, it appears that athletes’ appear to turn to other, more harmful, forms of escapism, such as alcohol, drugs, or suicide (e.g., Carlisle and Marshall, 2013). As Sarkar and Fletcher (2014) noted, more research is required to examine the interrelationships between psychological resilience, mental health, and wellbeing in elite athletes. However, rather than inaccurately labeling ‘mentally weak behaviors,’ such as denying stress or support, as a ‘dysfunctional side of resilience,’ sport psychologists should expose such myths so that psychosocially virtuous qualities are nurtured to enhance *both* performance and health.

## Conclusion

Through the analysis of autobiographies this study has enhanced understanding of elite athletes’ experiences of depression, and the implications of its interaction with sport performance. The findings display a two-way interaction, with depression having implications for performance, and performance having implications for depression. The athletes’ studied in this research were able to use sport as an escape for a short period of time, but eventually the pressure to perform and the need to succeed generated a fear of failure and emotional cost of failure that inevitably exacerbated their symptoms.

## Author Contributions

All authors listed, have made substantial, direct and intellectual contribution to the work, and approved it for publication.

## Conflict of Interest Statement

The authors declare that the research was conducted in the absence of any commercial or financial relationships that could be construed as a potential conflict of interest.
